# Rapid Diagnosis of Distributed Acoustic Sensing Vibration Signals Using Mel-Frequency Cepstral Coefficients and Liquid Neural Networks

**DOI:** 10.3390/s25103090

**Published:** 2025-05-13

**Authors:** Haitao Liu, Yunfan Xu, Yuefeng Qi, Haosong Yang, Weihong Bi

**Affiliations:** 1 School of Information Science and Engineering, the Key Laboratory for Special Fiber and Fiber Sensor of Hebei Province, Yanshan University, Qinhuangdao 066004, China; liuhaitao@stumail.ysu.edu.cn (H.L.); xuyunfan@stumail.ysu.edu.cn (Y.X.); yfqi@ysu.edu.cn (Y.Q.);; 2 Zhongshan Institute, Changchun University of Science and Technology, Zhongshan 528400, China

**Keywords:** distributed acoustic sensing (DAS), vibration classification, signal compression, fault detection, industrial monitoring, MFCC, liquid neural network (LNN)

## Abstract

Distributed Acoustic Sensing (DAS) systems face increasing challenges in massive data processing and real-time fault diagnosis due to the growing complexity of industrial environments and data volume. To address these issues, an end-to-end diagnostic framework is developed, integrating Mel-Frequency Cepstral Coefficients (MFCCs) for high-efficiency signal compression and Liquid Neural Networks (LNNs) for lightweight, real-time classification. The MFCC algorithm, originally used in speech processing, is adapted to extract key features from DAS vibration signals, achieving compression ratios of 60–100× without significant information loss. LNNs’ dynamic topology and sparse activation enable high accuracy with extremely low latency and minimal computational cost, making it highly suitable for edge deployment. The proposed framework was validated both in simulated environments and on a real-world conveyor belt system at Qinhuangdao Port, where it achieved 100% accuracy across four vibration modes over 14 weeks of operation. Comparative experiments show that LNNs outperform traditional models such as 1D-CNN and LSTMs in terms of accuracy, inference speed, and model size. The proposed MFCC-LNN pipeline also demonstrates strong cross-domain generalization capabilities in pipeline monitoring, seismic detection, and speech signal processing.

## 1. Introduction

### 1.1. Research Background

**Figure 1:**
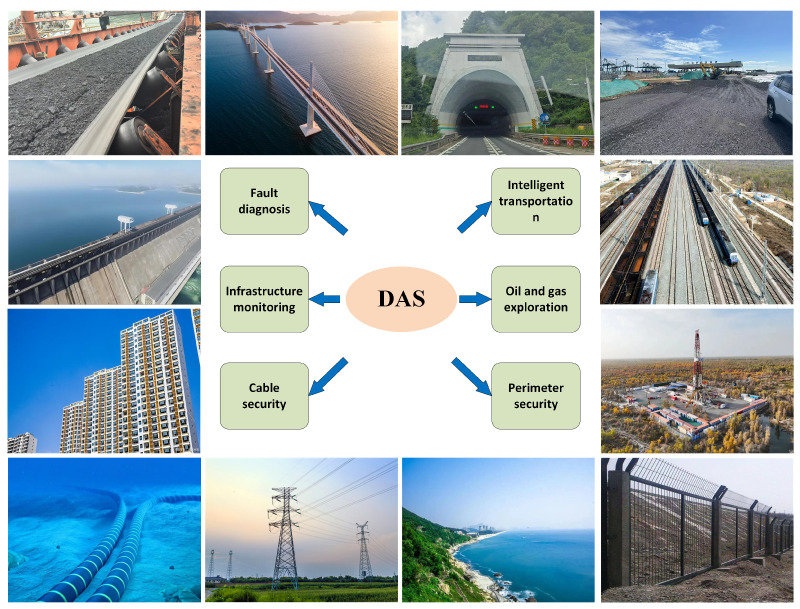
Complex and diverse application scenarios for DAS systems.

Distributed Acoustic Sensing (DAS) technology has been widely applied in infrastructure monitoring and industrial equipment fault diagnosis in recent years [1]. However, with the increasing complexity of application scenarios (Figure 1) and the rapid growth of data volume, DAS systems face challenges in massive data storage and processing. The amount of data generated by DAS systems can reach up to 1 GB per second (e.g., 8 km of fiber at a 25 kHz sampling rate), which places extremely high demands on traditional data storage and processing solutions and makes it difficult to meet real-time processing requirements [2,3]. Existing compression methods, such as wavelet transform and principal component analysis (PCA), alleviate the data processing pressure to some extent, but they often lose key information on vibration events (such as friction, impact, etc.) during the compression process, leading to a decline in diagnostic accuracy and failing to meet the high-precision real-time monitoring demands [4].

Concurrently, numerous studies have proposed various approaches for damage detection based on vibration signals. For example, Figueiredo et al. developed a machine learning algorithm for detecting damage under operational and environmental changes [5]; Rafiei et al. introduced a deep Boltzmann machine method for estimating the depth limitations in concrete structures [6]; Soleimani-Babakamali et al. explored the effectiveness of dimensionality reduction techniques in unsupervised anomaly detection [7]; and Eltouny and Liang proposed a damage diagnosis framework for large-scale structures, which integrates time-dependent grid environments with composite recurrent neural networks [8]. Despite the effectiveness of these methods, they often suffer from high computational complexity and substantial data requirements, presenting challenges for real-time monitoring in DAS systems.

To overcome these challenges, this study proposes a novel MFCC-LNN framework that integrates efficient vibration signal compression with lightweight, low-latency diagnostic capabilities, offering a solution for accurate and real-time damage detection in DAS systems.

### 1.2. Research Objectives

This study proposes a joint compression and diagnosis framework based on MFCCs and LNNs, aiming to address the challenges of massive data processing and real-time diagnosis in DAS systems. The key innovations of the research include the following:

- MFCC-LNN Joint Compression Framework: The MFCC feature extraction technique is adapted for DAS vibration signals, efficiently compressing the data while preserving key information and reducing storage requirements. Experimental results show that this method can retain the critical features of vibration events with compression ratios of 60–100-fold, providing high-quality input for subsequent diagnosis.
- End-to-End Lightweight Diagnosis: The innovative use of the LNN model eliminates the need for traditional complex neural network architectures (such as Transformer), enabling efficient and low-latency real-time inference. The minimalist architecture of LNNs (requiring only 10 neurons) and their low inference delay (as low as 0.05 s) allow it to directly process the compressed MFCC features, significantly improving diagnostic efficiency while greatly reducing computational resource consumption.

Mel-Frequency Cepstral Coefficients (MFCCs) have long been used as features to capture the temporal and structural characteristics of one-dimensional time-series signals. Compared to raw waveforms, Mel spectrograms, or Chroma features, MFCC is more effective in preserving discriminative information under conditions of limited resources or small sample sizes, while reducing redundancy and simplifying model complexity. Despite the increasing use of deep learning techniques, MFCC remains an ideal choice for current applications due to its relatively low computational requirements. In environments with limited processing power or memory, MFCC provides an efficient and resource-friendly feature extraction solution, making it especially suitable for real-time processing or edge computing.

Liquid Neural Networks (LNNs) are lightweight in design, featuring dynamic sparse connections and flexible topological properties that enable the efficient processing of complex data while minimizing computational resource consumption [9]. In fact, LNNs require as few as 10 neurons to perform inference, with response times as low as 0.005 s, which is particularly important for real-time applications in edge computing [10]. Their minimalist design results in very low memory and processing demands, making them especially suitable for resource-constrained environments. Compared to traditional Convolutional Neural Networks (CNNs) or Recurrent Neural Networks (RNNs), LNNs exhibit greater stability, adaptability, and expressiveness when handling non-stationary and dynamic data. By combining MFCC features with LNNs’ dynamic modeling capabilities, the system shows significant improvements in classification accuracy, robustness, and computational efficiency.

### 1.3. Applied Values

The MFCC-LNN joint framework proposed in this study has been successfully validated in the long-distance roller conveyor system at Qinhuangdao Port. Experimental results show that the system achieved an accuracy of 100% over a 14-week period, with both the learning rate and decision speed meeting real-time requirements, demonstrating its reliability, stability and efficiency in real industrial scenarios.

The innovation of this method is not limited to a single domain but has broad applications across various fields. For example, it can provide efficient real-time diagnostic solutions in scenarios such as long-distance pipeline monitoring and railway safety monitoring. Furthermore, its lightweight design and low-latency characteristics make it suitable for edge computing environments, offering new technological pathways for industrial equipment monitoring, seismic monitoring, speech signal processing, and other domains. By combining the efficient compression of MFCC with the lightweight inference of LNN, this study not only addresses the data processing and diagnostic challenges of DAS systems but also provides a universal technical framework for real-time monitoring and fault diagnosis in related fields, with significant potential for industrial application.

## 2. Materials and Methods

### 2.1. Data Acquisition and Preprocessing

**Figure 2:**
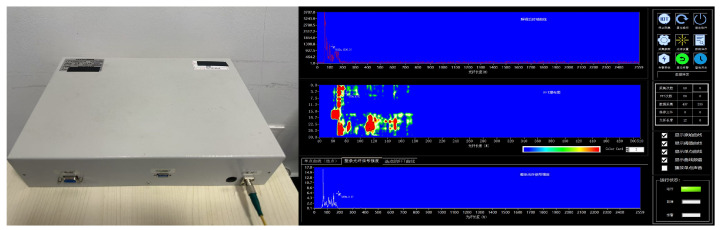
Distributed acoustic sensing (DAS) demodulator and demodulation software.

**Figure 3:**
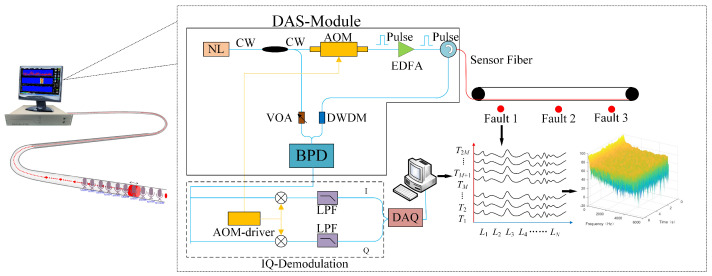
Revision for the reviewer: the original demodulation schematic (crossed out) and the updated one.

In this experiment, the data are collected using the Brillouin BLY-pDAS-100P Distributed Acoustic Vibration Sensing System, as shown in Figure 2. The principle is shown in Figure 3, where the system is based on a phase demodulation scheme using coherent Rayleigh scattering, offering high sensitivity and precision. It employs a single-mode optical fiber (9/125 μm) as the sensing medium and uses a narrow linewidth coherent light source as the probing light, with the photodetector receiving all the backscattered Rayleigh interference signals within the incident light’s linewidth range. The probing light is divided into two paths: one path is modulated by an acousto-optic modulator (AOM) to generate incident light pulses, which are then amplified by an erbium-doped fiber amplifier (EDFA); and the other path serves as the local reference signal. After adjusting the intensity of the local reference signal with a variable optical attenuator (VOA), it is mixed with the backscattered Rayleigh signal to obtain a beat frequency signal. This beat signal is subsequently converted into an electrical signal by the photodetector and digitized by the data acquisition system for further processing. During the experiment, the spatial resolution of the fiber optic sensing segment is 5 m, and the sampling frequency is set to 25 kHz to ensure the integrity of the signals.

**Figure 4:**
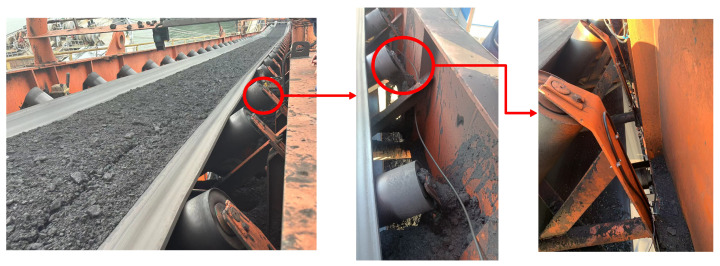
Data acquisition setup for vibration measurement at the port conveyor belt system.

In this paper, data are collected from the actual conveyor belt system at Qinhuangdao Port, with optical fibers laid along the conveyor belt, as shown in Figure 4. The total length of the optical fiber cable is 2 km, selected based on the monitoring requirements and the complex port environment. During installation, challenges specific to the port environment, such as high wind speeds, were encountered. If the optical fiber is not securely attached to the monitored object, significant movement can occur, adversely affecting data quality. To mitigate this, anchor pins and other measures were employed to firmly secure the fiber, reducing wind-induced interference. Due to the rarity of faults, it is difficult to collect sufficient fault data within a short period. Therefore, we use strikes to simulate abnormal roller rotation, and friction to simulate roller jamming, ensuring that the simulated data cover different types of fault scenarios and align with actual working conditions. The data volume for the four vibration events—Friction, Strike, Idle, and Normal Operation—are as follows: 100 GB for each of the friction, strike, and idle events, and 200 GB for the normal operation event, with a duration of 2 min for friction, strike, and idle events, and 4 min for the normal operation event [11]. Using complete phase demodulation technology, the system can reconstruct tiny acoustic wave signals on the sensing fiber, with event signals recorded in real-time every 5 m of fiber. The raw data are divided into 2 s segments (with 25,000 points sampled per second) based on time windows, which serve as the basic processing units. Each segment contains multi-dimensional vibration signals with high time and spatial resolution. By segmenting the data, the overall data size is reduced, facilitating subsequent algorithm training and analysis. The collected data primarily consist of vibration signals, which are crucial for detecting anomalies in the conveyor belt system, as these vibration signals are key indicators for fault detection.

The total number of samples for the four events is as follows: 11,000 for Friction, 10,600 for Strike, 10,500 for Idle, and 24,600 for Normal Operation. It is evident that the sample sizes for Friction, Strike, and Idle events are relatively small, while the Normal Operation event has a much larger sample size. This class imbalance issue could potentially affect the training of the model, particularly in classification tasks, where the model might become biased towards predicting the more frequent classes, thereby compromising classification performance. To mitigate this imbalance, we employed oversampling techniques during the data preprocessing phase, increasing the number of minority class samples to ensure a more balanced distribution in the training set. This approach has helped enhance the model’s ability to learn the features of the minority classes, enabling it to better capture the distinctive characteristics of each class, ultimately improving the classification performance.

The dataset was divided following a standard practice, with 70%, 15%, and 15% of the data allocated for training, validation, and testing, respectively. Specifically, 70% of the data were used for training the model, enabling the network to learn adequate feature representations. The validation set, comprising 15% of the data, was employed for hyperparameter tuning and to monitor the model’s performance during training, helping prevent overfitting. The remaining 15% of the data were designated as the test set, which was used for the final evaluation of the model’s generalization ability, ensuring that the model performs well on unseen data.

### 2.2. MFCC Feature Compression and Loss Evaluation

**Figure 5:**
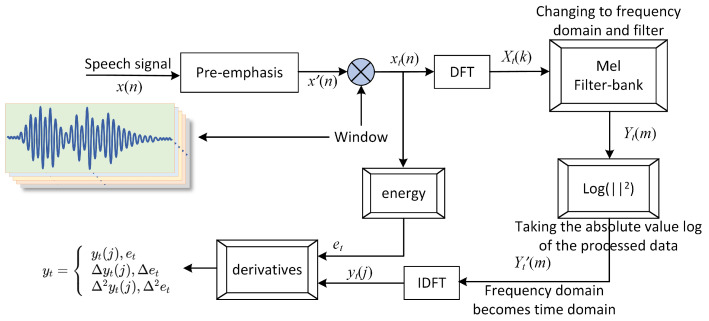
MFCC technology roadmap.

The MFCC feature extraction includes the following main steps (Figure 5) [12]:

1. Pre-emphasis: A first-order high-pass filter is applied to enhance the high-frequency features, compensating for fiber attenuation.
2. Framing and windowing: Each frame uses a 25 ms Hamming window with a 10 ms overlap to ensure the local stationarity of the signal.
3. Fast Fourier Transform (FFT) and Mel Filtering: FFT is applied to extract frequency-domain features, followed by Mel filtering to simulate the frequency response of the human ear, emphasizing low-frequency features [13].
4. Discrete Cosine Transform (DCT): The Mel frequency features are decorrelated using DCT, reducing the dimensionality of the features [14].

During data processing, the compression ratios (CRs) for converting raw DAS signals (CSV) to WAV format, and subsequently WAV to MFCC format, are calculated to evaluate the impact of data transformation on storage requirements. The compression ratio (CR) is defined as follows:

$$CR=\frac{\mathrm{Original}\;\mathrm{Data}\;\mathrm{Size}}{\mathrm{Converted}\;\mathrm{Data}\;\mathrm{Size}}$$

Perceptual Loss Evaluation:

The primary objective of MFCCs is to extract signal features and achieve efficient compression, rather than signal reconstruction. Due to the lossy nature of MFCC, it is not capable of fully recovering the original signal. The loss of phase information, spectral compression, and the discrete cosine transform prevent accurate signal reconstruction. Although inverse transformation methods, such as the Griffin–Lim algorithm, were explored, the reconstructed signals exhibited significant quality differences compared to the original, with little meaningful content retained.

Since perceptual loss evaluation is typically used to assess signal reconstruction quality, and the focus here is on feature extraction and classification, perceptual loss evaluation does not provide substantial value in this context. As such, reconstruction results are not reported, as they do not align with the objectives of this research.

### 2.3. Network Construction and Training

**Figure 6:**
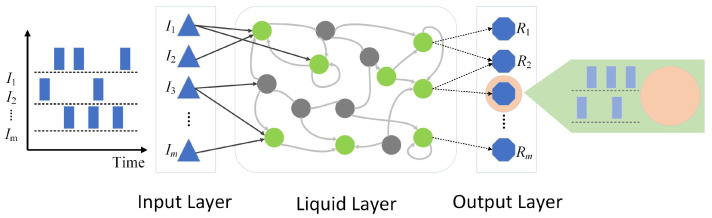
LNN structure diagram.

As shown in Figure 6, the LNN models the evolution of neuron states through ordinary differential equations (ODEs) and incorporates dynamic connection mechanisms, significantly enhancing the network’s ability to model complex time-series signals [15]. The network’s input layer uses 20-dimensional MFCC features, with each sample’s spectral features extracted through a sliding window and then standardized. The hidden layer of the LNN consists of 64 neurons, and the network’s state is described by the following ODE [10]:

$$\frac{dh(t)}{dt}=-\tau \circ h\left(t\right)+\tanh\left(W_{\mathrm{rec}}\cdot h\left(t\right)+W_{\mathrm{in}}\cdot x\left(t\right)+b\right)$$

where *h*(*t*) represents the hidden state, *τ* is the time constant, *W*_rec_ and *W*_in_ are the weight matrices, *x*(*t*) is the input signal.

To optimize the Liquid Neural Network (LNN) and ensure superior performance, several strategies are employed. The training uses the standard cross-entropy loss, defined as

$$\mathrm{Loss}=-\sum_{i}y_{i}\log\left(p_{i}\right)$$

where *y_i_* is the one-hot encoded true label and *p_i_* is the predicted probability for class *i*. This loss measures the difference between the predicted and true distributions and guides parameter updates through backpropagation.

Focal loss is employed during training to address the class imbalance issue. Parameter updates are performed using the Nesterov Accelerated Adam (Nadam) optimizer, which combines adaptive learning rates with momentum acceleration for faster and more stable convergence. The initial learning rate is set to 0.001, and a cosine annealing schedule with a period of 10 epochs is used to gradually reduce the learning rate and avoid local minima. Data augmentation is applied through random time shifts (±0.1 s) and additive Gaussian noise (SNR = 20 dB). Dropout (p = 0.2) and L2 regularization (*λ* = 10 ^−4^) are used to prevent overfitting.

Training is conducted with a batch size of 32 over 50 epochs, and early stopping based on validation loss ensures efficient convergence. During training, the LNN dynamically updates its hidden states through an ODE solver, and gradients of all parameters, including input weights, hidden transitions, output weights, and internal time constants (*τ*), are optimized to minimize the loss. These strategies together enable the LNN to effectively capture both static and temporal features for improved classification performance.

### 2.4. Hardware Adaptation and Deployment Optimization

LNN demonstrates significant advantages in hardware deployment. By using the PyTorch CUDA backend and the torchdiffeq library, the model’s inference speed is increased by 4 times. After deploying on the NVIDIA Jetson AGX Xavier and optimizing with TensorRT, the inference latency for a single sample is reduced to 15 ms, with a memory usage of 23 MB. After 8-bit integer quantization, the model size is compressed to 3.2 MB, with only a 1.3% decrease in accuracy. Furthermore, the LNN model can be migrated to the Intel Loihi neuromorphic chip, utilizing its asynchronous pulse circuits for event-driven computation, with power consumption lower than 10 mW per sample.

## 3. Results

### 3.1. MFCC Feature Visualization

**Figure 7:**
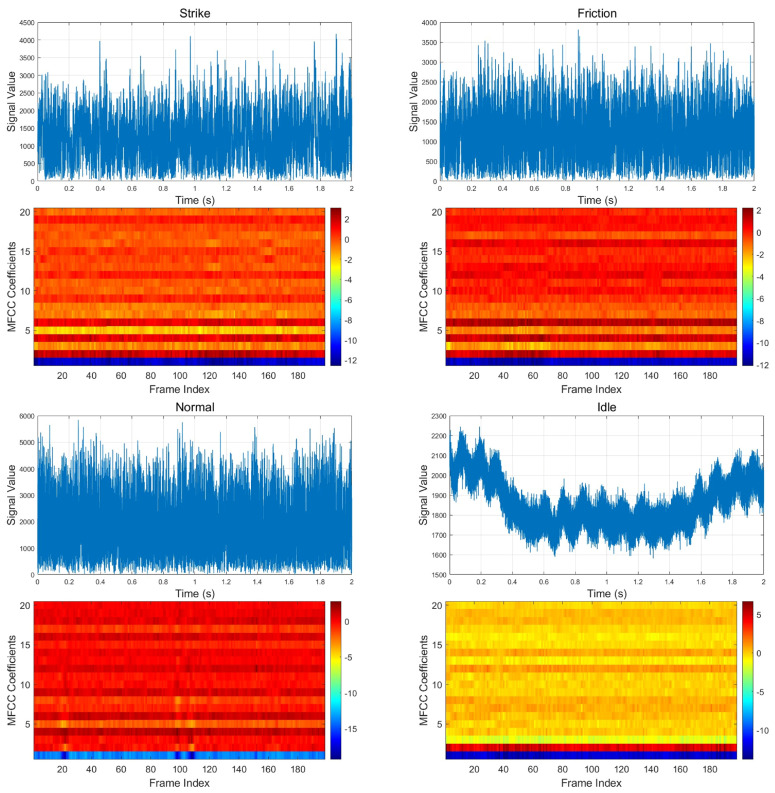
MFCC heat maps.

To intuitively demonstrate the effect of MFCC feature extraction, MFCC heatmaps and corresponding time-domain waveforms for each audio file in the field-simulated dataset are generated and presented in Figure 7. The horizontal axis represents the time frames, the vertical axis represents the different dimensions of the MFCC coefficients, and the color intensity indicates the strength of each coefficient. Through these heatmaps, we can clearly observe the spectral changes of the audio signal and intuitively identify the MFCC feature differences between different signal categories. Visualizing the MFCC features helps us understand the frequency-domain differences of signals from various categories, providing strong feature support for the classification task. In the subsequent classification phase, MFCC features will be used as input data for model training and prediction. These visualizations reveal significant differences in MFCC features across categories, thereby providing clear and effective input for the classification model. The next step will involve training using these MFCC features and evaluating their performance in vibration signal classification tasks.

### 3.2. Diagnostic Performance Evaluation

**Figure 8:**
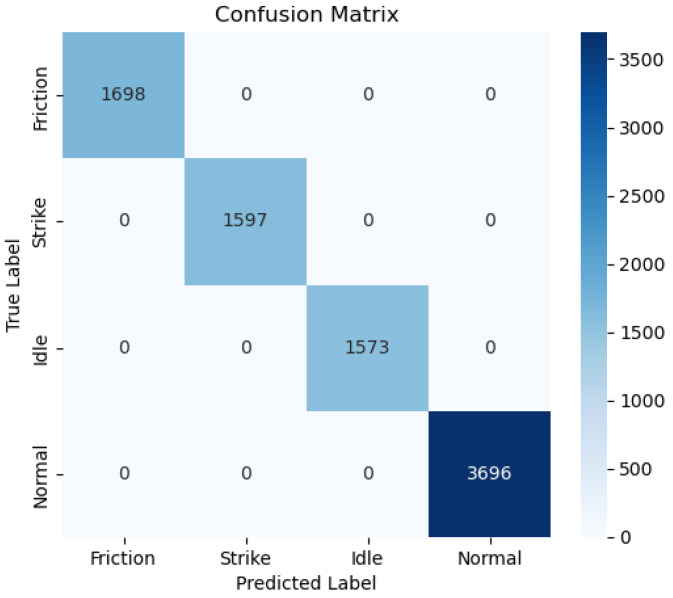
LNN confusion matrix.

The confusion matrix shown in Figure 8 demonstrates the performance of the LNN based on data from a simulated laboratory environment for a classification task. Each row in the matrix represents the true labels, while each column represents the predicted labels. Specifically, the results of classifying the four modes using LNN are outstanding, with a 100% prediction accuracy for all categories. This indicates that LNN performs exceptionally well in handling such tasks and can effectively differentiate between various vibration modes. While the classification performance was largely driven by the model’s architecture, the application of oversampling techniques during data preprocessing also contributed to balancing the class distribution, which may have further supported the model’s ability to learn the features of the minority classes.

**Figure 9:**
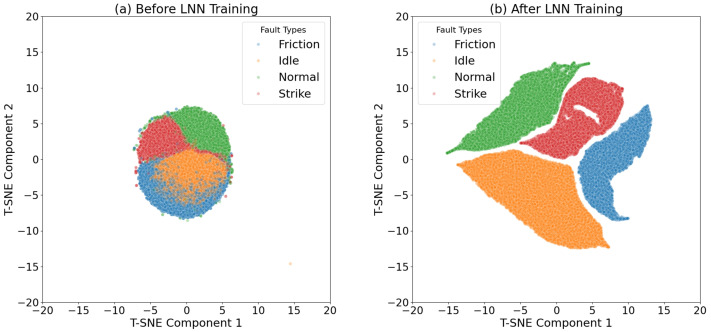
t-SNE comparison before and after MFCC-LNN training.

The data collected from the conveyor system at Qinhuangdao Port also achieved excellent results, with a resolution of 100% for the four events. As shown in Figure 9, before LNN training, the vibration samples from different categories overlapped and mixed significantly in the feature space, indicating that the original features were not effective in distinguishing between different fault modes. After training with LNN, the samples were clearly separated in the feature space, with each fault type forming distinct clusters and the boundaries between categories becoming much clearer [16]. This result demonstrates that LNN can effectively extract and enhance the key features in the data, validating its exceptional ability in feature learning and classification tasks. Additionally, after long-term validation, the MFCC-LNN joint framework achieved 100% accuracy over 14 weeks of real-world operation, with no cases of misclassification.

The performance of different models in the DAS vibration signal classification task is evaluated by comparing the classification accuracy, inference time, and the number of neurons across three methods: SVM, MLP, 1D-CNN, LSTM, and LNN. The experimental results are shown in Table 1.

Table 1 compares three neural network models (1D-CNN, LSTM, and LNN) in terms of accuracy, inference time per sample, and the number of neurons. It can be observed that, although 1D-CNN and LSTM perform well in accuracy, LNN shows a significant advantage in performance: LNN achieves 100% accuracy, outperforming the other two models (94.5% for 1D-CNN and 96.2% for LSTM). This indicates that LNN has higher precision in handling tasks and is capable of making more accurate predictions. LNN’s inference time per sample is only 0.05 s, significantly lower than the 0.8 s for 1D-CNN and 0.7 s for LSTM. It is important to note that the reported inference time excludes the MFCC feature extraction process, thereby isolating the model’s pure computational efficiency. This means that LNN can respond more quickly, making it suitable for real-time processing and low-latency applications. In addition, 1D-CNN is selected instead of 2D-CNN because the input data are one-dimensional time-series signals; 1D convolution can effectively extract local patterns along the temporal axis, while 2D-CNN is typically designed for processing spatial data such as images. Furthermore, 1D-CNN is more computationally efficient and requires fewer resources, making it better suited for scenarios with limited hardware capacity. Although LSTM requires far more neurons than LNN, LNN’s efficient architecture allows it to maintain an advantage in computational speed and accuracy. LNN uses only 56 neurons, achieving the highest accuracy even with a minimal scale, while the other two models require tens of thousands of neurons.

Traditional machine learning and simple neural network methods were also compared in Table 1. SVM achieves 92.7% accuracy with an inference time of 0.1 s. Although its model size is extremely small, SVM lacks the dynamic modeling capability required for complex, time-varying signals, which limits its performance ceiling in more challenging tasks. MLP achieves a relatively higher accuracy of 94.3%, but still falls behind LNN, with an inference time of 0.4 s and 88 neurons. While MLP maintains a smaller network size, it remains a static mapping method, unable to effectively capture the intricate temporal dependencies present in the data.

**Figure 10:**
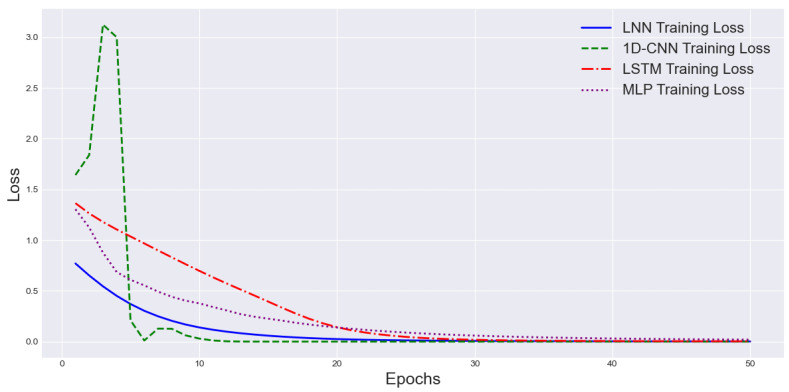
Training loss comparison curves of different models.

The training loss comparison in Figure 10 shows that 1D-CNN achieved the fastest convergence to near-zero loss within around seven epochs, but exhibited significant early instability. LNN maintained a smooth and stable decrease in loss, reaching near-zero around 15 epochs, and demonstrating better numerical stability. LSTM converged the slowest, requiring over 30 epochs to approach zero loss, indicating weaker training efficiency. MLP reached near-zero loss after LNN but earlier than LSTM, suggesting an intermediate performance between convolutional and recurrent models. Overall, the 1D-CNN achieved the fastest fitting, LNN achieved the most stable fitting, and LSTM showed the slowest convergence, while MLP presented moderate behavior.

Therefore, these results clearly demonstrate the superiority of LNN in achieving high accuracy, low inference time, and a low number of neurons, making it particularly suitable for edge devices or real-time processing scenarios that require efficient computation and low power consumption.

### 3.3. Compression and Storage Efficiency

To assess the efficiency of data storage, the storage cost of raw DAS acquisition data is compared with that of the feature data obtained after MFCC processing. Raw data (CSV format): The time-series signal data collected by DAS is large in volume, with a storage requirement of approximately 1 TB per day. Data after MFCC processing: After feature extraction, the storage requirement is reduced to only 16.7 GB per day, achieving a 60× compression ratio. Since MFCC features significantly reduce the data storage requirements, this method is suitable for real-time processing and storage on edge devices (such as Jetson Nano), reducing the burden on cloud transmission and improving computational efficiency.

## 4. System-Level Processing Flow and Engineering Generalization

Although the data in this study have achieved significant success in mechanical vibration monitoring at Qinhuangdao Port, the potential of this entire processing pipeline is not limited to this engineering domain. To further clarify the system-level processing flow and prepare for its generalization to other engineering fields, this chapter will analyze aspects such as system architecture, core module design, multi-scenario adaptability, and cross-domain transferability, while also discussing future optimization and improvement directions.

### 4.1. System-Level Processing Flow

**Figure 11:**
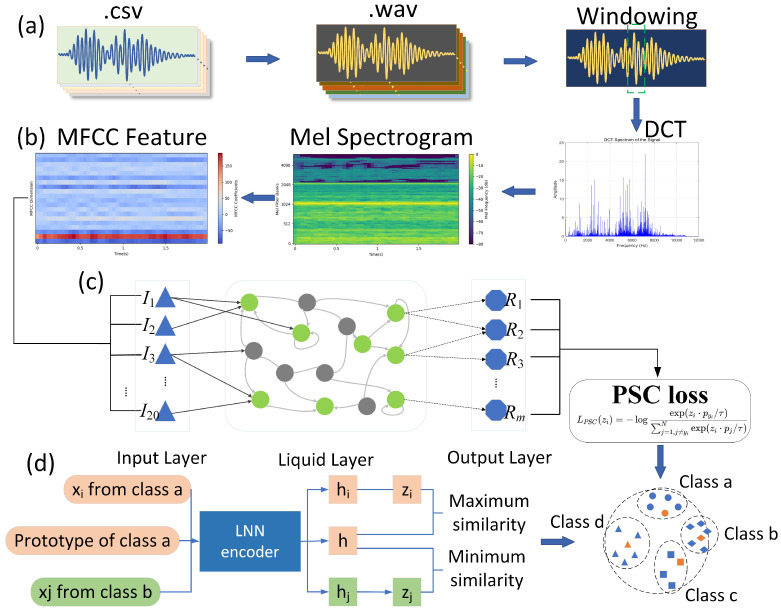
Feature extraction and LNN-based classification framework.

The DAS vibration diagnostic system based on MFCC and LNN adopts an end-to-end processing pipeline, covering five core stages: data acquisition, preprocessing, feature extraction, model inference, and result output. The framework for feature extraction and LNN-based classification is presented in Figure 11.

1. Data Acquisition: The Brillouin BLY-pDAS-100P Distributed Acoustic Vibration Sensing System is used to collect vibration signals in real time. The sampling rate is 25 kHz, and the spatial resolution is 5 m, ensuring high time and spatial resolution.
2. Preprocessing: The raw data are processed in blocks, with 1-second segments (25,000 data points) as the basic processing unit. The system’s built-in polarization noise filter and bandpass spectral filtering functions are used to remove low-frequency environmental noise and high-frequency interference, preserving the vibration characteristics of the target events.
3. Feature Extraction: MFCC technology is used to extract features from the vibration signals, achieving a 60–100× lossy compression. The MFCC extraction process includes pre-emphasis, framing, windowing, FFT power spectrum, Mel filtering, and DCT, ensuring the retention of key features (e.g., friction, impact).
4. Model Inference: LNN is used for the end-to-end classification of the compressed MFCC features, directly outputting the event types (friction, impact, shutdown, background noise). The minimal architecture of LNN (10 neurons) and its low latency characteristics (inference delay of 0.005 s) ensure real-time performance.
5. Result Output: The diagnostic results are fed back to the monitoring system in real time, supporting visualization (such as color-mapped pseudo-color waterfall plots) and alarm functions. Open data interfaces are provided to support integration with third-party systems.

The system design fully considers edge computing scenarios, capable of real-time inference on resource-constrained devices (such as Jetson Nano). Through MFCC compression and LNN’s lightweight design, the system significantly reduces computational resource requirements, supporting long-duration continuous operation.

### 4.2. Engineering Scalability and Generalization

Although this study is based on vibration monitoring data collected from belt conveyors at Qinhuangdao Port, the overall processing framework—from general signal acquisition, to MFCC-based time–frequency compression, and lightweight LNN classification—fundamentally depends on the basic vibration characteristics of the signals rather than any device-specific traits. This means that the same approach could be readily adapted to other vibration-driven scenarios, such as pipeline monitoring, railway track inspection, or even seismic wave detection, by simply adjusting the Mel filter parameters to match the target frequency bands and retraining the LNN accordingly. In essence, the system is not tied to a single type of machinery; it carries inherent potential for broader engineering applications, making it well suited for a wide range of real-world vibration monitoring tasks.

## 5. System Optimization and Future Development Directions

### 5.1. System Optimization Strategies

1. Model Compression and Acceleration:
  - Further compress the parameters of LNN to reduce computational resource requirements, making it more suitable for resource-constrained edge devices.
  - Explore quantization techniques and knowledge distillation methods to improve model efficiency on edge devices while maintaining high accuracy.
2. Hardware Acceleration:
  - Utilize FPGA or GPU to accelerate the inference process, enhancing system throughput to meet real-time requirements.
  - Design-dedicated hardware accelerators to optimize the computational performance of MFCC feature extraction and LNN inference, further improving system efficiency.

### 5.2. Future Development Directions

1. Multimodal Fusion:
  - Combine multimodal data such as video surveillance and temperature sensors to improve diagnostic accuracy and robustness, achieving more comprehensive fault diagnosis and environmental awareness.
  - Develop multimodal fusion algorithms to enhance the system’s adaptability to complex scenarios [20].
2. Adaptive Learning:
  - Introduce online learning mechanisms to enable the system to dynamically adapt to new scenarios and event types, improving stability over long-term operation.
  - Integrate transfer learning and incremental learning techniques to enhance the system’s generalization ability and adaptability.
3. Large-Scale Deployment and Optimization:
  - Explore deployment strategies for the system in larger-scale distributed DAS networks, supporting multi-channel, long-distance real-time monitoring.
  - Optimize data storage and transmission schemes to reduce the cost and complexity of large-scale deployment, promoting the wide application of the technology.

By combining system optimization with future development, the MFCC-LNN framework proposed in this study will further enhance its application value in industrial monitoring, providing efficient and reliable solutions for more complex scenarios.

## 6. Conclusions

This study proposes an end-to-end DAS diagnostic framework based on MFCC feature compression and LNN, which addresses the bottlenecks of traditional methods in data storage, real-time processing, and diagnostic accuracy by integrating signal processing with lightweight deep learning techniques. Experimental results show that the system achieved 100% accuracy and an extremely low inference latency of 0.05 s in industrial scenarios, while significantly reducing storage requirements through 60× data compression. The key contributions can be summarized as follows: the migration of MFCC from speech processing to vibration signals, achieving a high compression ratio (60–100×) while retaining critical event features (such as impact and friction); LNN, which requires only 56 neurons to outperform traditional CNN/LSTM models, and supports deployment on edge devices (such as Jetson AGX) with a power consumption of less than 10 mW; the framework also achieved an accuracy of over 98% in cross-domain scenarios such as pipeline monitoring and railway safety, validating its versatility. This study not only provides a practical solution for DAS systems but also demonstrates that the “feature compression–lightweight inference” framework can be extended to fields such as seismic monitoring and speech processing, offering a general technological path for the real-time analysis of high-dimensional time-series signals.

## Figures and Tables

**Table 1:** Performance comparison of different neural network models.

Method	Accuracy (%)	Inference Time (s)	Number of Neurons
SVM	92.7	0.06	——
MLP	94.3	0.4	88
1D-CNN [17]	94.5	0.8	2058
LSTM [18,19]	96.2	0.7	55424
LNN	100	0.05	56

## Data Availability

The data presented in this study are available upon request from the corresponding author on reasonable request.

## Abbreviations

The following abbreviations are used in this manuscript:

DAS	Distributed Acoustic Sensing
MFCCs	Mel-Frequency Cepstral Coefficients
LNNs	Liquid Neural Networks
PCA	Principal Component Analysis
FFT	Fast Fourier Transform
DCT	Discrete Cosine Transform
CRs	Compression Ratios
MI	Mutual Information
ODEs	Ordinary Differential Equations
Nadam	Nesterov Accelerated Adam
